# A pyridyl-benzopyridoindole-dione probe for ultrafast fluorometric selective detection of diethyl chlorophosphate (DCP) nerve agent mimic with latent fingerprint and logic gate applications

**DOI:** 10.1039/d6ra08860k

**Published:** 2026-11-09

**Authors:** Anju Ranolia, Laxmi Narayan, Anil Duhan, Sudhir Kumar, Parvin Kumar, Devender Singh, Ahmad Umar, Sotirios Baskoutas, Jayant Sindhu

**Affiliations:** a Department of Chemistry, Chaudhary Charan Singh Haryana Agricultural University Hisar-125004 Haryana India jayantchem@gmail.com; b Department of Bioinformatics and Computational Biology, Chaudhary Charan Singh Haryana Agricultural University Hisar-125004 Haryana India; c Department of Chemistry, Kurukshetra University Kurukshetra-136119 Haryana India parvinjangra@gmail.com; d Department of Chemistry, Maharshi Dayanand University Rohtak-124001 Haryana India; e Department of Chemistry, College of Science and Arts, and STEM Pioneers Training Lab, Najran University Najran-11001 Kingdom of Saudi Arabia; f Department of Materials Science and Engineering, The Ohio State University Columbus 43210 OH USA; g Department of Materials Science, University of Patras Patras Greece

## Abstract

Organophosphorus nerve agents pose severe threats to public health and national security, driving the urgent need for rapid, sensitive and field-deployable detection system. In the present work, a pyridyl benzopyridoindole dione (PBID) fluorophore featuring naphthoquinone core as an intramolecular charge-transfer (ICT) modulator is reported for selective and sensitive fluorescence “*turn-off*” detection of diethyl chlorophosphate (DCP). DCP addition caused five-fold emission quenching at 615 nm with a visual orange-to-lavender color change under UV light (365 nm). The response is attributed to pyridine nitrogen phosphorylation followed by hydrolysis to protonated PBID, as supported by ^1^H NMR titration and DFT calculations. The probe enables ultrafast detection within 15 s, achieving limit of detection (LOD) and quantification (LOQ) values of 0.67 and 2.24 µM, respectively. Beyond solution-phase sensing, PBID was successfully translated into practical platforms, including paper-based test strips, solid-state reversible sensing, latent fingerprint (LFP) visualization on non-porous surfaces and smartphone-integrated RGB colorimetric analysis for on-site quantification. PBID also enabled an "IMPLICATION" molecular logic gate and showed reliable DCP recovery from environmental water and soil samples. An Analytical GREEnness (AGREE) metric score of 0.80 further supports the sustainability of the method.

## Abstract 2

A pyridyl benzopyridoindole dione (PBID) fluorophore enables ultrafast and selective detection of diethylchlorophosphate. The PBID platform integrates solution, vapor phase sensing, solid-state reversibility and environmental sample recovery of DCP.

## Graphical abstract

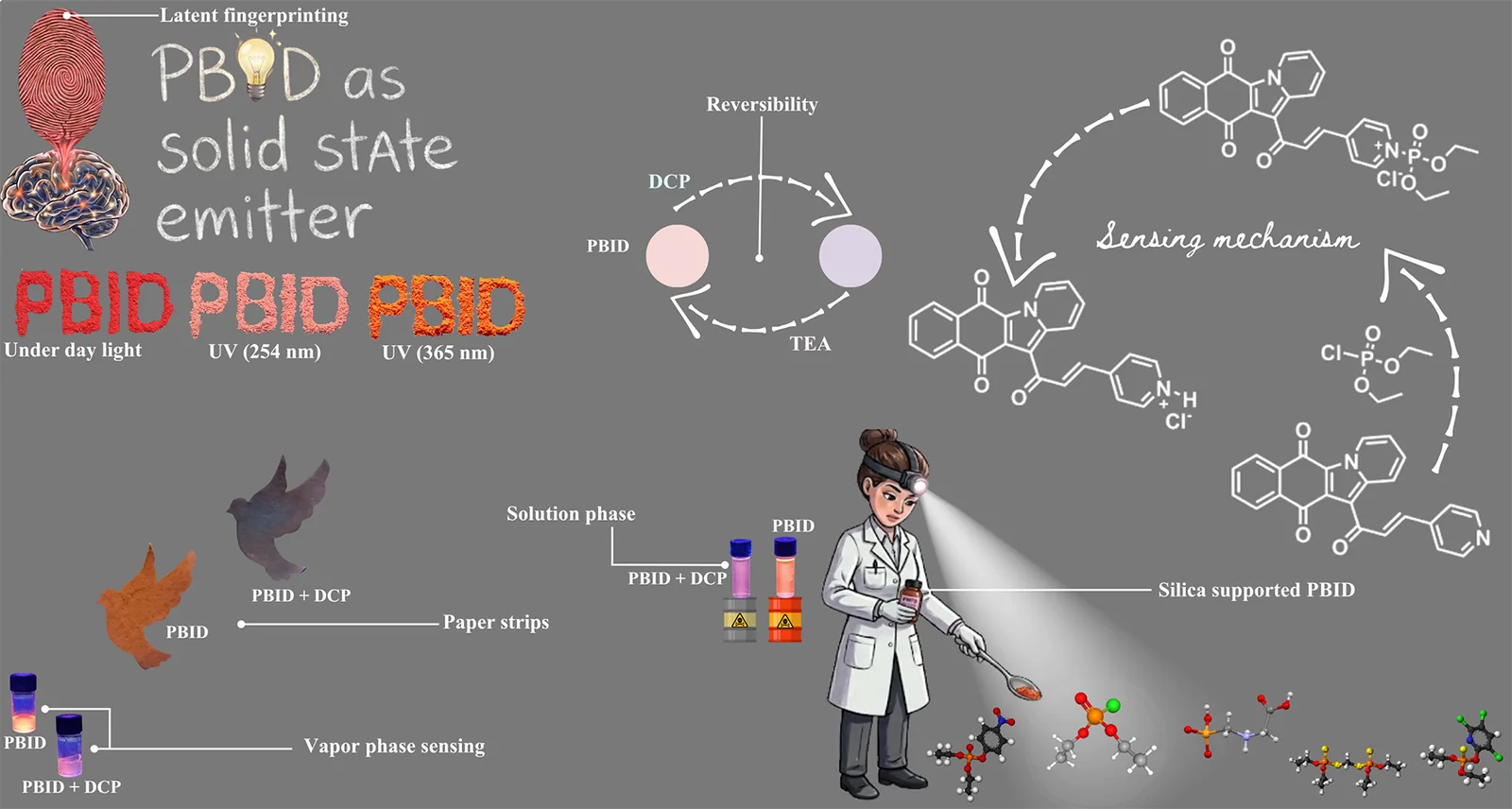

## 1. Introduction

Nerve agents are lethal neurotoxic organophosphorus compounds that can instantly induce fatal consequences by irreversibly disrupting neurological actions.^1–7^ Based on their structural features, physicochemical characteristics and toxicological effects, nerve agents are generally grouped into V-series, G-series and Novichok agents (Fig. 1).^8–11^ Tabun (GA), Sarin (GB), Soman (GD) and Cyclosarin (GF) are G-series agents known for their high volatility, easy environmental dispersion and severe neurotoxic effects.^12,13^ Their devastating potential has been demonstrated in warfare, terrorism and assassination attempts including the Iran-Iraq war, Tokyo attack, sarin deployment during the Syrian civil war, VX-mediated assassination of Kim Jong-nam and Novichok-related poisoning in Salisbury, UK.^11,14–25^ These incidents highlighted the persistent threat posed by nerve agents to civilian populations, military personnel and global security.

**Fig. 1:**
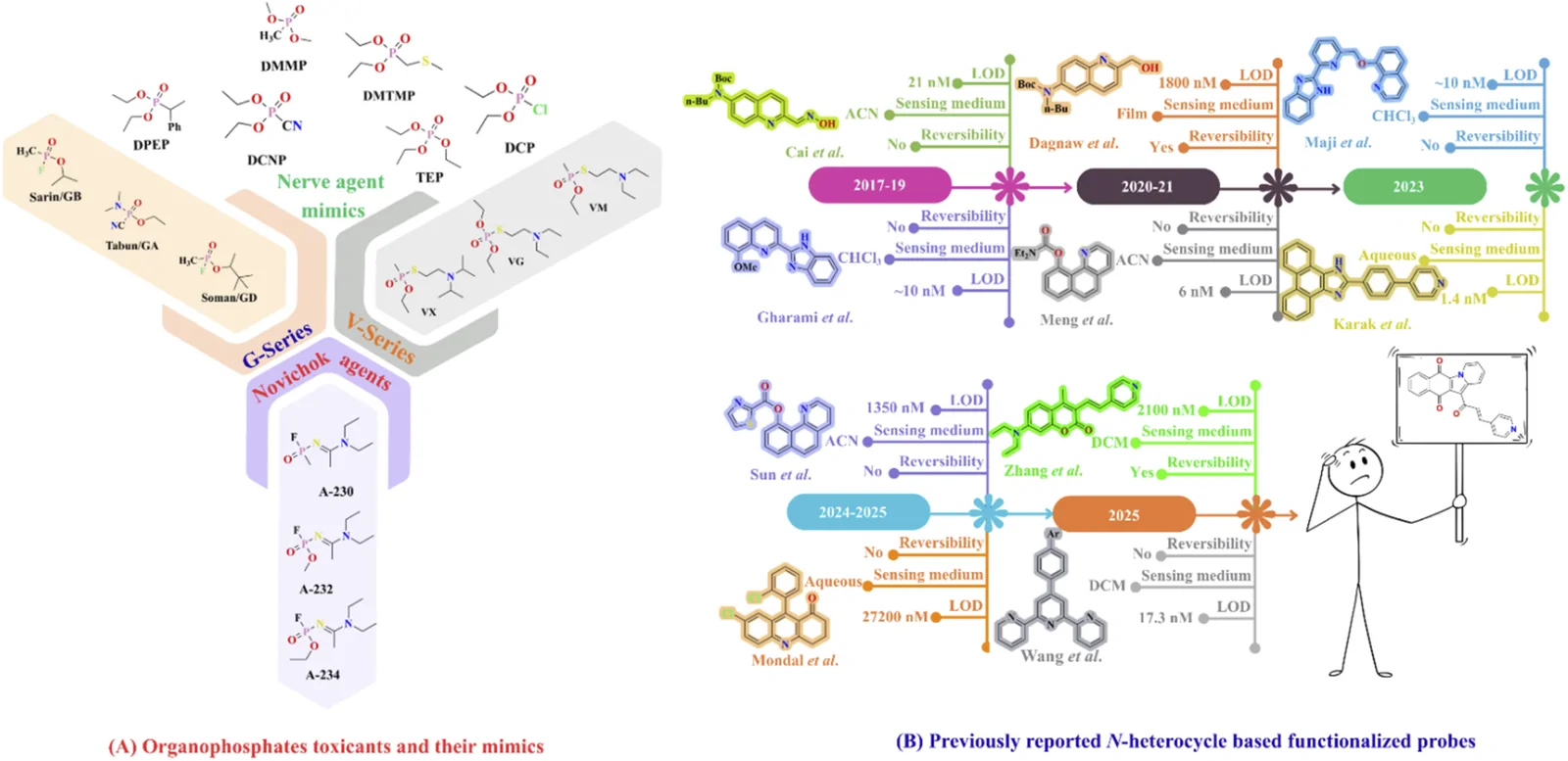
(A) Organophosphates toxicants and their mimics and (B) comparison of the performance of previously reported N-heterocycle based functionalized probes.

Mechanistically, nerve agents exert toxicity by covalently phosphorylating acetylcholinesterase, leading to enzyme inhibition and overstimulation of acetylcholine at the synaptic cleft.^26–29^ This overstimulation causes neuromuscular paralysis, respiratory failure and ultimately death.^30–32^ Beyond their use as chemical warfare agents, synthetic organophosphorus pesticides are widely applied in agricultural practices for pest control, leading to the contamination of soil, groundwater and aquatic environments.^20^ Exposure through contaminated water, food and bioaccumulated aquatic organisms, even at trace ppm levels, may cause neurotoxic effects under both acute and long-term exposure.^5,23^ Structurally related simulants of nerve agents contain highly reactive phosphorylating centre and suitable leaving groups, which support their use as analyte in developing sensing platforms.^12,21,22^ Therefore, the development of rapid, selective and field deployable detection platforms for organophosphorus toxicants remain critically important.^33,34^

Several conventional methods, including interferometry-based sensor, lanthanide-based luminescence probes, electrochemical based sensor, gas chromatography, high-performance liquid chromatography (HPLC) and nanomaterial-assisted sensors have been reported for detecting these neurotoxins and other potential analytes.^7,35–39^ However, many of these approaches require sophisticated instrumentation, trained personnel and longer response times. Fluorescence-based detection offers a promising alternative due to its high sensitivity, selectivity, direct visual output, rapid response and minimal infrastructure requirement.^1,7,26,31,40–44^

Because of strict safety regulations and international restrictions associated with nerve agents, research investigations often rely on less hazardous alternatives, such as diethyl chlorophosphate (DCP), as a surrogate for sarin.^45^

In this study, sarin (GB) was considered as the target nerve agent of interest; however, DCP was used as a safer and chemically relevant model compound because it preserves important physicochemical features of GB.^46^ Several heteroatom based nucleophilic recognition sites have been explored in the development of fluorescent probes for DCP detection.^2,6,43,45,47–50^ Compared with previously reported N-heterocycle functionalized probes, the present probe PBID exhibits superior analytical performance by overcoming limitations such as higher detection limit, restricted solvent compatibility and lack of reversible sensing behavior.^6,28,33,51^ In this context, we designed and synthesized pyridyl benzopyridoindole dione (PBID) as a conjugated fluorophore for the selective and robust recognition of diethylchlorophosphate (DCP).

## 2. Experimental details

### 2.1 Materials and methods

2,3-Dichloronaphthoquinone was purchased from Thermo-Fisher and all other building blocks were obtained from Sigma-Aldrich and used as such. Similarly, organic and inorganic organophosphates such as DCP, TEP, TBP, DMMP, DCMP, DEPA, Na_2_HPO_4_, KH_2_PO_4_, glyphosate, acephate, isoproturon, paraoxon, ethion, chlorothalonil, chlorphyrifos and azamethiphos were also procured from Sigma-Aldrich. All NMR related experiments were performed in DMSO-*d*_6_ on NMR spectrophotometer (400 MHz) from Jeol. The FT-IR spectra was recorded on Thermo-Scientific Nicolet iS and LC-MS analysis was performed on Waters Micromass Q-Tof Micro (ESI) spectrometer. All the photophysical studies were carried out using Shimadzu UV-1900i and PerkinElmer 6500 spectrophotometers.

### 2.2 Synthesis protocol of (*E*)-12-(3-(pyridin-4-yl)acryloyl)benzo[*f*]pyrido[1,2-*a*]indole-6,11-dione (PBID)

A mixture of 2,3-dichloronapthoquinone (1) (0.022 mmol), acetyl acetone (2) (0.11 mmol) and pyridine (3) (0.11 mmol) in methanol (10 mL) was refluxed for 30 min to afford the desired 12-acetylbenzo[*f*]pyrido[1,2-*a*]indole-6,11-dione (ABID). Subsequently, a solution of ABID (1 mmol) in MeOH was reacted with pyridine-4-carboxaldehyde (4, 1.3 mmol) *via* Claisen–Schmidt reaction in round bottomed flask (100 mL) using NaOH (10%) as base. The content of the reaction flask was continuously stirred at ambient temperature for 12 h and continuous reaction monitoring was performed with pre-coated thin layer chromatography (TLC) plates (Merck) using EA : Hexane (3 : 7, v/v) as the mobile phase (Scheme 1). Upon complete consumption of reactants, a solid precipitates during the reaction, which was filtered and recrystallized by MeOH to afford analytically pure orange-colored PBID in 80% yield. A % atom economy of 73.8 indicates the greenness of synthetic methodology. The final structure of PBID was confirmed using FT-IR, NMR and mass spectrometry (Fig. S6–S8, SI).

**Scheme 1:**
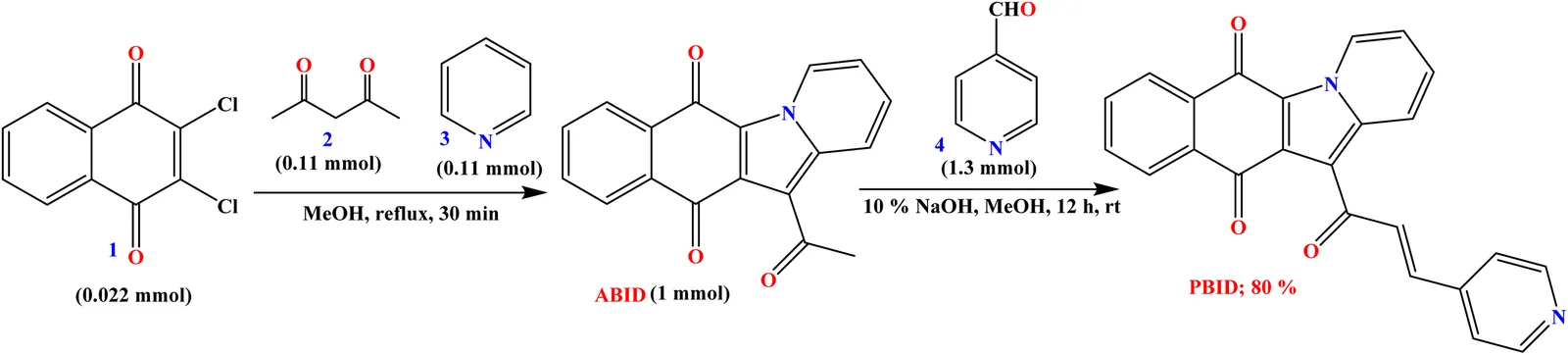
Sequential approach for the synthesis of (*E*)-12-(3-(pyridin-4-yl)acryloyl)benzo[*f*]pyrido[1,2-*a*]indole-6,11-dione (PBID).

### 2.3 Spectral details

Vibrant red solid; M. pt: 248–250 °C; yield: 80%; *R*_f_ (EtOAc : hexane (30 : 70, v/v)) = 0.375; ^1^H NMR (400 MHz, DMSO-*d*_6_): *δ*_H_ 9.73–9.71 (m, 1H), 8.60 (d, *J* = 6.0, 2H), 8.24–8.22 (m, 1H), 8.13–8.07 (m, 2H), 7.87–7.76 (m, 3H), 7.66–7.61 (m, 3H), 7.56 (d, *J* = 16 Hz, 1H), 7.45–7.40 (m, 1H); ^13^C NMR (100 MHz, DMSO-*d*_6_): *δ*_C_ 187.04, 182.18, 150.91, 142.78, 137.98, 134.79, 134.04, 132.29, 130.49, 128.58, 127.46, 126.31, 122.78, 120.87, 119.45; mass spectra: *m*/*z* calculated 379.1012 [M + H]^+^; found 379.1072 [M + H]^+^.

### 2.4 Spectral studies

A stock solution of 1 mM was prepared for PBID by dissolving its 3.7 mg in 10 mL of DMSO. Similarly, a 0.01 M stock solution of organophosphates including DCP, TEP, TBP, DMMP, DCNP and DEPA, along with inorganic phosphates Na_2_HPO_4_ and KH_2_PO_4_ were prepared in CH_3_CN (10 mL). The stock solutions for glyphosate, acephate, isoproturon, paraoxon, ethion, chlorthioanil, chlorpyrifos and azamethiphos were also prepared in CH_3_CN at a concentration of 50 mM. UV-Vis absorption spectra were recorded between 300–600 nm while the emission profile was recorded in the range of 563–620 nm. The excitation wavelength used for fluorescence experiment was 478 nm. The slit width for both excitation and emission was kept at 10 nm, with a PMT gain of 8× and an applied voltage of 400 V.

### 2.5 Theoretical calculations

The ground state geometry optimization of PBID and PBID–DCP complex was performed using a combination of B3LYP/6-311+G(d,p) level based density functional method. Optimized geometries of PBID and its DCP bound complex were confirmed by frequency calculations, as no imaginary frequencies were observed. All the calculations were performed using the Gaussian 16 package,^52^ and the results were visualized using Avogadro 2.0.^53^

### 2.6 Soil extraction procedure

Different type of soil, including sandy loam, sandy, black and clay loamy were utilized for detection of DCP. A known concentration of DCP (60 µM) was spiked into all the soil samples and then air-dried. After allowing them to stand for 2 h, chloroform was added to the soil samples. The content of the beaker was then filtered to separate soil from chloroform. The chloroform containing DCP solution was then evaporated over rota-evaporator. The crude thus left was dissolved in MeOH and the dissolved portion was then mixed with PBID (20 µM) solution.^20,54^

### 2.7 Collection of fingerprints

#### 2.7.1 Donor consent

Latent fingerprints were collected from one of the authors, who voluntarily provided the samples. The donor gave informed consent for the collection and use of the fingerprints in this study. No personal identifying information was recorded or reported.

Prior to fingerprint deposition, the fingertip was first washed with distilled water and allowed to dry completely. The finger was then gently pressed onto the selected substrates for 2–5 s under mild pressure. During fingerprint deposition, residues naturally present on the fingertip including sweat and sebaceous secretions were transferred onto the substrate surface. This resulted in the formation of latent fingerprints that were not visible under normal observation. To improve consistency among samples, the contact duration and applied pressure were kept as uniform as possible during the deposition process on different substrates.

#### 2.7.2 Development of latent fingerprinting (LFPs) by powder dusting method

A small amount of PBID was applied on feather brush and then carefully applied with soft and controlled strokes over the substrate bearing latent fingerprints (LPFs). This allowed the PBID particles to adhere preferentially to the fingerprint residues thereby enhancing the ridge patterns for visualization. Excess powder was removed by gently blowing air blower. Care was taken during powder application to prevent over-deposition, ensuring that the ridge details remained clearly visible. The developed LFPs were then visualized and photographed under UV light at 365 nm, where clear fluorescent fingerprint patterns could be observed.

## 3. Results and discussion

### 3.1 Rationale of design

Rational molecular design is a crucial aspect in the development of efficient chemosensors, as the sensing performance strongly depends on the precise arrangement of electronic, structural and reactive units within the probe. In particular, π-conjugated systems are attractive because extended electronic delocalization can enhance absorption, fluorescence emission and analyte responsive optical behaviour. Previously, we demonstrated that structural modulation of the molecular framework can play an important role in achieving selective analyte recognition. In our earlier work, the parent acetyl based scaffold was post-functionalised into a hydrazone unit, which enabled selective DCP recognition through a PET-based sensing mechanism.^20^ However, this design mainly introduced a recognition site and showed comparatively lower emission wavelength due to limited electronic conjugation. In a subsequent study, the conjugation length was extended through Knoevenagel condensation, leading to an extended framework with higher emission wavelength that was successfully applied for selective cyanide sensing.^55^

Based on these findings, the parent work further modulates the molecular framework through Claisen–Schmidt condensation to construct a donor–π–acceptor (D–π–A) conjugated framework for the selective detection of DCP. The acetyl based framework acts as an electron-rich donor (push unit), while the pyridine ring acts as an electron-deficient acceptor (pull unit) interconnected through a π-conjugated bridge (Fig. 2). This push–pull mechanism improved photo-physical properties by promoting π-delocalization and charge transfer. Upon exposure to DCP, the nucleophilic pyridine nitrogen interacts with the electrophilic phosphorus centre, which disrupts the charge-transfer process and results in a fluorescence “*turn-off*” response.

**Fig. 2:**
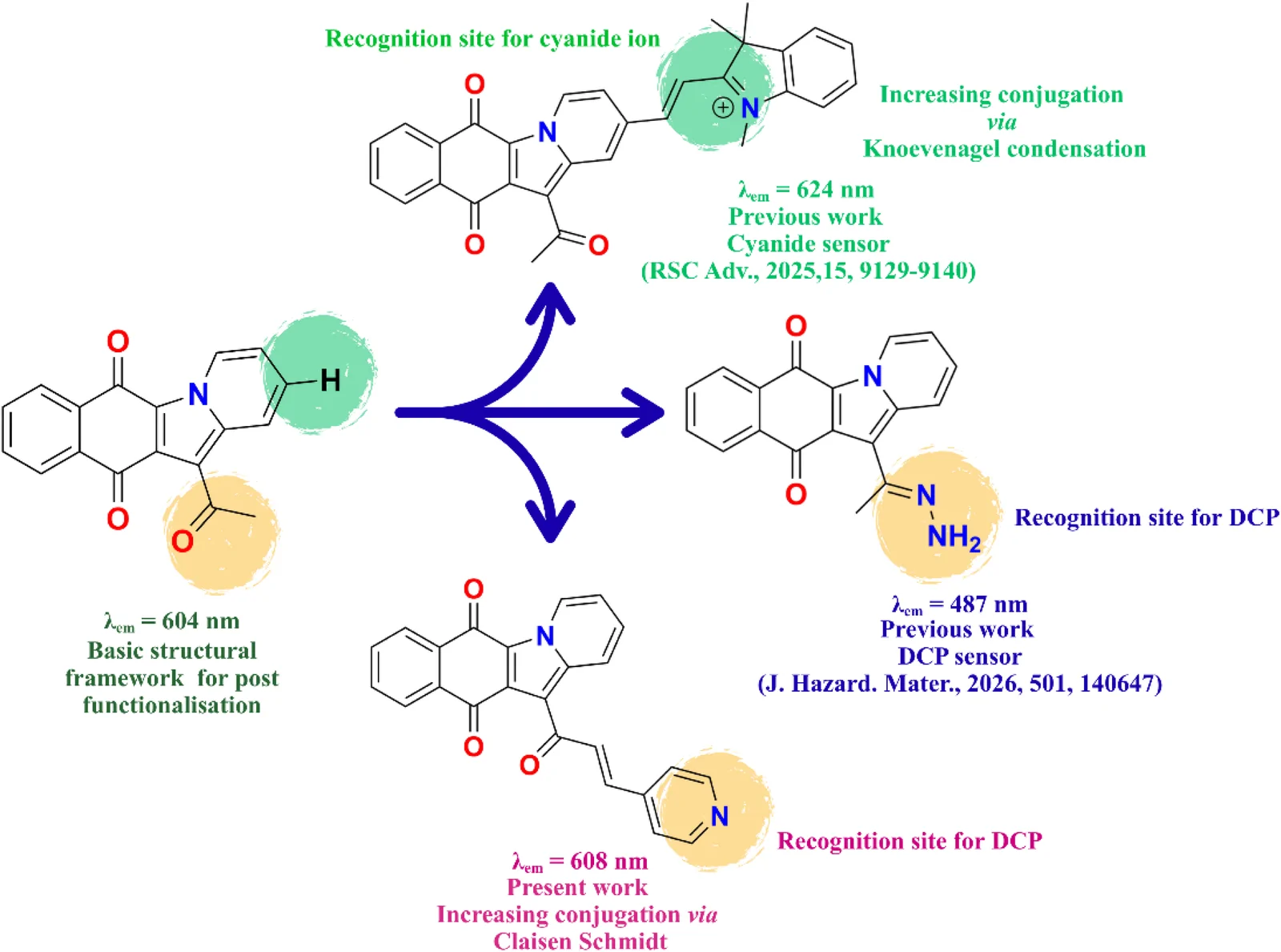
Rationale of designing (*E*)-12-(3-(pyridin-4-yl)acryloyl)benzo[*f*]pyrido[1,2-*a*]indole-6,11-dione (PBID).

### 3.2 Solvatochromic response

PBID was systematically utilized for its solvatochromic behavior by recording its photophysical response in solvents of varying polarity, including H_2_O, DMSO, ACN, DMF, MeOH, DCM, CHCl_3_ and Glycerol (Fig. 3). Its absorption spectra displayed a prominent band between 475–512 nm due to π–π* transition. A clear solvent dependent variation in the absorption maxima was observed, with a hypsochromic (blue) shift in relatively polar solvents compared with less-polar chlorinated solvents such as DCM and CHCl_3_. This indicates that the polar environment stabilizes ground state more efficiently over excited state, resulting in negative solvatochromism.

**Fig. 3:**
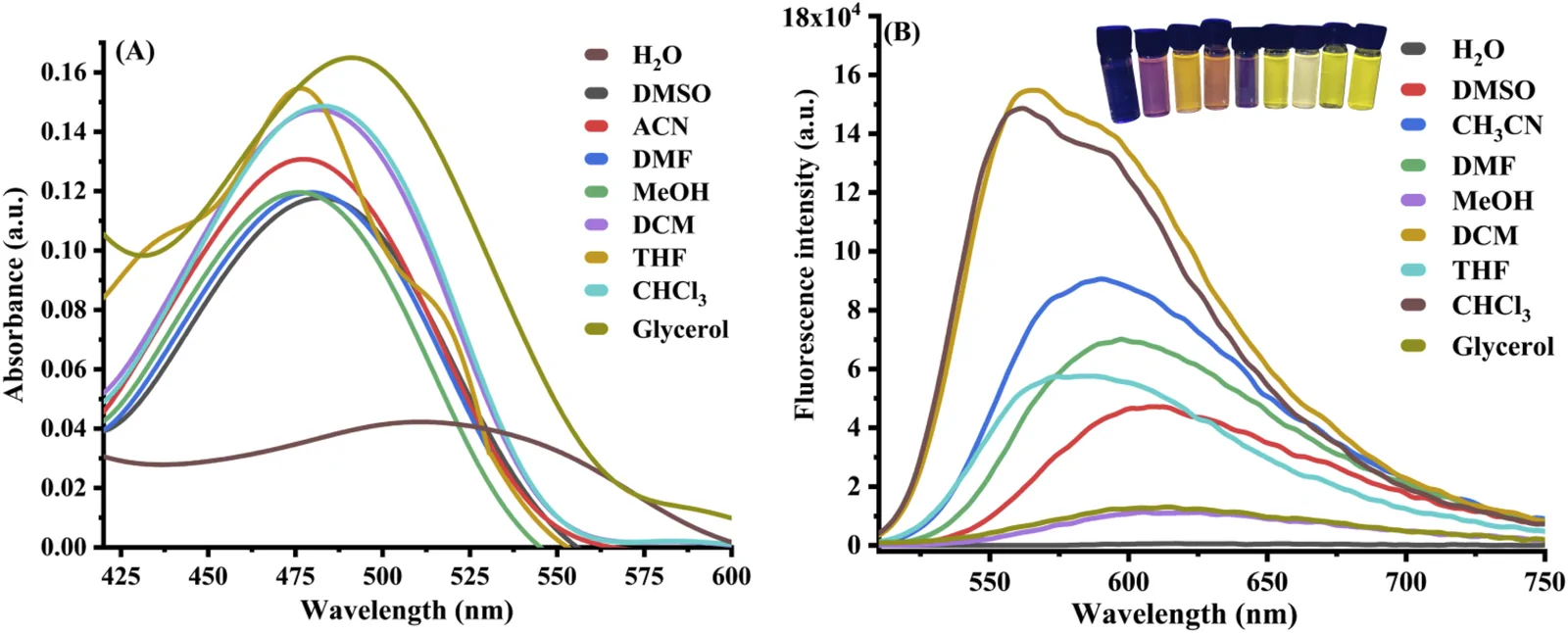
(A) Absorbance and (B) emission spectra of PBID in solvents of varying polarity.

In contrast, the fluorescence spectra of PBID recorded in the range of 563–620 nm, displayed an opposite trend compared with its absorption spectra (Fig. 4B). In case of polar aprotic solvent such as DMSO, the emission band was observed around 608 nm. In polar-protic solvent such as H_2_O and MeOH, a bathochromic shift was observed, with the emission maxima appearing at 615–620 nm. Overall, in protic solvents, a pronounced bathochromic (red) shift was observed, which indicates greater stabilization of the excited state likely due to enhanced dipole–dipole interactions and possible intramolecular charge transfer (ICT) effect in polar media.^56,57^

**Fig. 4:**
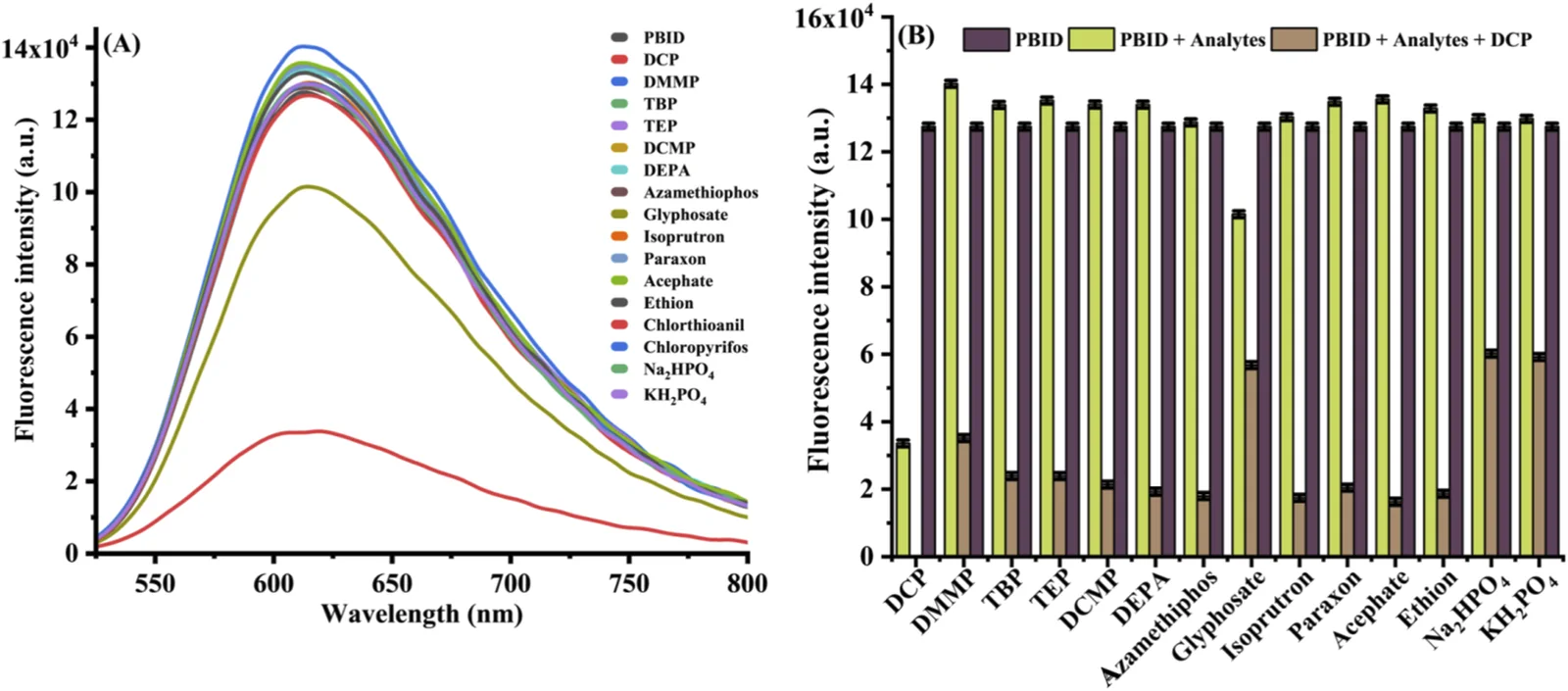
(A) Fluorescence based sensing of organophosphates (20 µM) with PBID solution (20 µM) in MeOH and (B) interference based studies in presence of various analytes.

Stokes shift is an important parameter expressed in cm^−1^ that provides insight into the excited-state behaviour of a fluorescent probe. It represents the difference between the absorption and emission maxima, indicating the degree of relaxation that occurs in the excited-state. In the present study, the largest Stokes shift was observed in MeOH, which may be attributed to strong solvent–solute interactions and enhanced stabilization of the excited state (Fig. S1). Furthermore, the Lippert–Mataga analysis provides insight into the solvent dependent changes in dipole moment between the ground and excited states (Fig. S1). The plot of Stokes shift *vs.* orientation polarizability (Δ*f*) exhibited a good linear correlation with an *R*^2^ value of 0.87.

### 3.3 Optical response of PBID towards various analytes

MeOH was selected as the medium for subsequent sensing studies, as it displayed maximum Stokes shift. The sensing performance of PBID was systematically investigated against wide range of analytes including DCP, TEP, TBP, DMMP, DCNP, DEPA, Na_2_HPO_4_, KH_2_PO_4_, glyphosate, acephate, isoproturon, paraoxon, ethion, chlorthioanil, chlorpyrifos and azamethiphos in MeOH (Fig. 4A). PBID displayed a strong emission maxima centered at 615 nm, indicating its inherent emissive nature. Upon addition of DCP, a “turn-off” response was observed, suggesting the formation of PBID–DCP complex. The color of the solution shifted from orange-yellowish to lavender when visualized under UV light (365 nm). In comparison, the remaining analytes showed no appreciable change in the emission intensity. However, glyphosate produced only a slight decrease in emission intensity, with a quenching effect much weaker than that observed for DCP. These results suggest that PBID exhibits strong selectivity toward DCP in the presence of other tested analytes. To confirm this selectivity, interference experiments were carried out using mixtures containing different competing ions. DCP maintained its characteristic fluorescence quenching response even in mixed-analyte system, supporting its preferential interaction with PBID (Fig. 4B). Although minor interference was observed from glyphosate and inorganic phosphates such as Na_2_HPO_4_ and KH_2_PO_4_. Overall, the outcomes demonstrate that PBID is a highly selective and reliable probe for DCP detection with minimal cross-reactivity, making it a promising candidate for practical sensing applications in complex environment.

### 3.4 Titration profile and quantitative parameters

To evaluate other analytical parameters, fluorescence titration studies were performed by the incremental addition of DCP over a concentration range of 0–20 µM (Fig. 5A). A systematic reduction in the emission intensity with increasing DCP concentration, confirming the fluorescence quenching response. The emission intensity displayed a good linear correlation with DCP concentration over a concentration range of 0–18 µM, highlighting the applicability of probe PBID for quantitative analysis. The limit of detection (LOD) and quantification (LOQ) were calculated to be 0.67 and 2.24 µM, respectively, demonstrating the high sensitivity of PBID for trace-level detection of DCP (Fig. 5B).

**Fig. 5:**
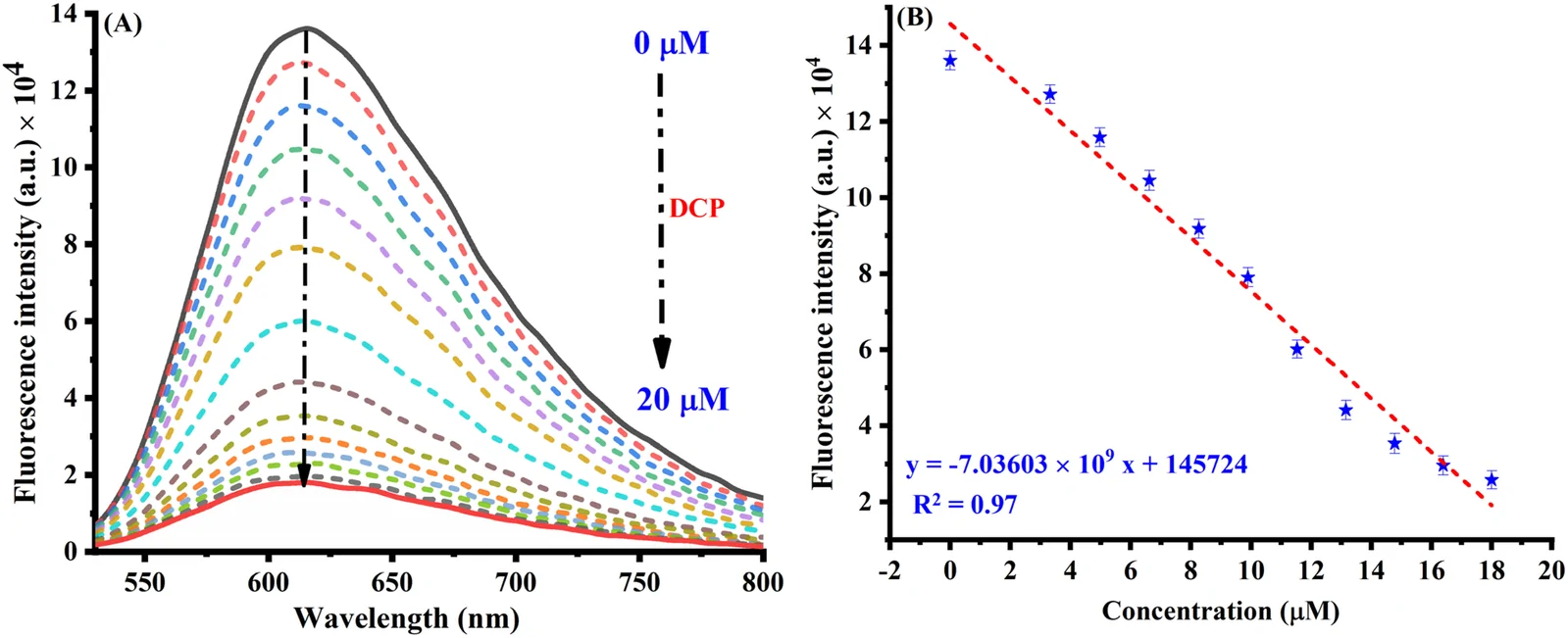
(A) Titration profile of PBID (20 µM) in the presence of DCP (0–20 µM) and (B) a linear calibration curve (0–18 µM) for LOD and LOQ.

The binding stoichiometry between PBID and DCP was found to be 1 : 1 (Fig. S2). Overall, these results indicate the formation of a stable complex and support the sensing performance of PBID towards DCP.

### 3.5 Time-dependent fluorescence spectra and reversibility profile

Response time is an important analytical parameter, as it measures how quickly a sensing system responds to the target species.^20^ Upon addition of DCP to PBID solution, a rapid decrease in the fluorescence intensity was observed within 15 s (Fig. 6A). This fast quenching response indicates the high responsiveness of PBID and supports its practical applicability as an efficient sensing platform. In addition, the reversibility of PBID was also examined to evaluate its reusability and alignment with the 3R principles of reduce, reuse and recycle.^58^

**Fig. 6:**
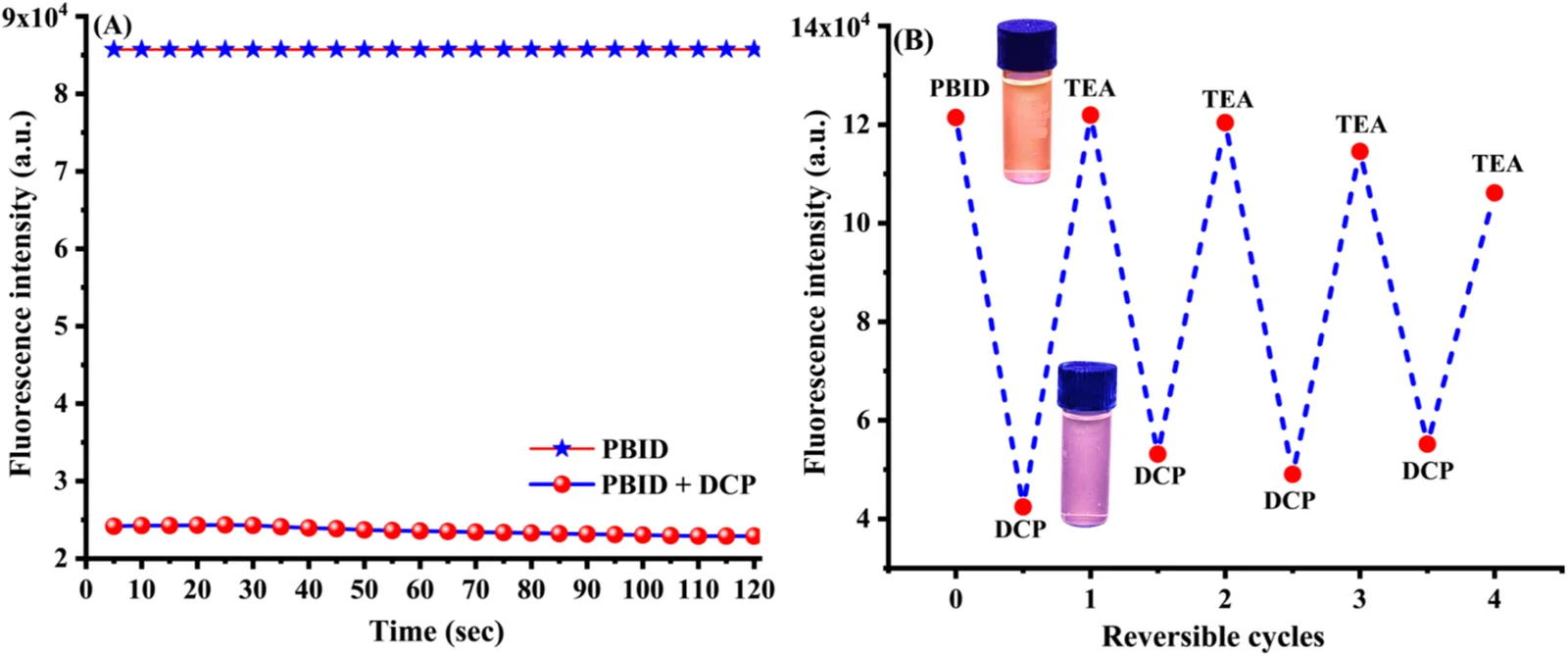
(A) Time-dependent profile of PBID and PBID–DCP complex and (B) reversibility pattern of PBID–DCP complex in presence of TEA.

The ability of probe to return to its original state after interaction with the analyte demonstrated its reusability and reducing material consumption, thereby supporting the “Reduce” aspect. This regenerative capability may also reduce waste generation and overall operational cost, aligning well with the “Recycle” concept. The reversibility of the PBID–DCP system was systematically examined in the presence of triethyl amine (TEA). Upon addition of DCP (20 µM) to the PBID solution (20 µM), a rapid decrease in the emission intensity was observed, indicating a “*turn-off*” response. However, subsequent addition of TEA (20 µM) restored the original signal, and this response was successfully for repeated up to four cycles. This reusability may reduce material use and improve the cost-effectiveness of PBID for real-time sensing applications (Fig. 6B).

### 3.6 Photo-stability and thermal-stability

The photo-stability of PBID and its DCP bound complex was evaluated under continuous irradiation of UV light (365 nm) for a duration of 1 h. Negligible loss in the emission intensity was noticed when the spectra were recorded at a regular interval of 15 min, indicating strong resistance to photo-bleaching and suitability for prolonged imaging (Fig. S3). The thermal stability of PBID and its DCP bound complex was also examined over a wide temperature range (20–45 °C) (Fig. S3). The fluorescence response remained almost unchanged, with only minimal variation in the emission intensity, indicating the reliable performance of PBID under different environmental and experimental conditions.

### 3.7 Plausible sensing mechanism

After establishing the photo-physical behavior of PBID, a plausible sensing mechanism was proposed using results of ^1^H NMR titration and DFT calculations. The formation of a PBID–DCP complex was proposed by ^1^H NMR titration. PBID exhibited characteristic peak at *δ*_H_ 9.71 ppm, corresponding to the proton *ortho* to the nitrogen atom of the indolizine ring. Similarly, the *ortho* proton of the pyridine ring are assigned at *δ*_H_ 8.61 ppm. After addition of DCP, these resonances shifted downfield to *δ*_H_ 9.78 and 8.78 ppm, respectively. This shift may be attributed to the protonation of the pyridine nitrogen following the hydrolysis of the phosphorylated intermediate (Fig. 7). These NMR changes provide a strong evidence of the interaction of PBID with DCP.

**Fig. 7:**
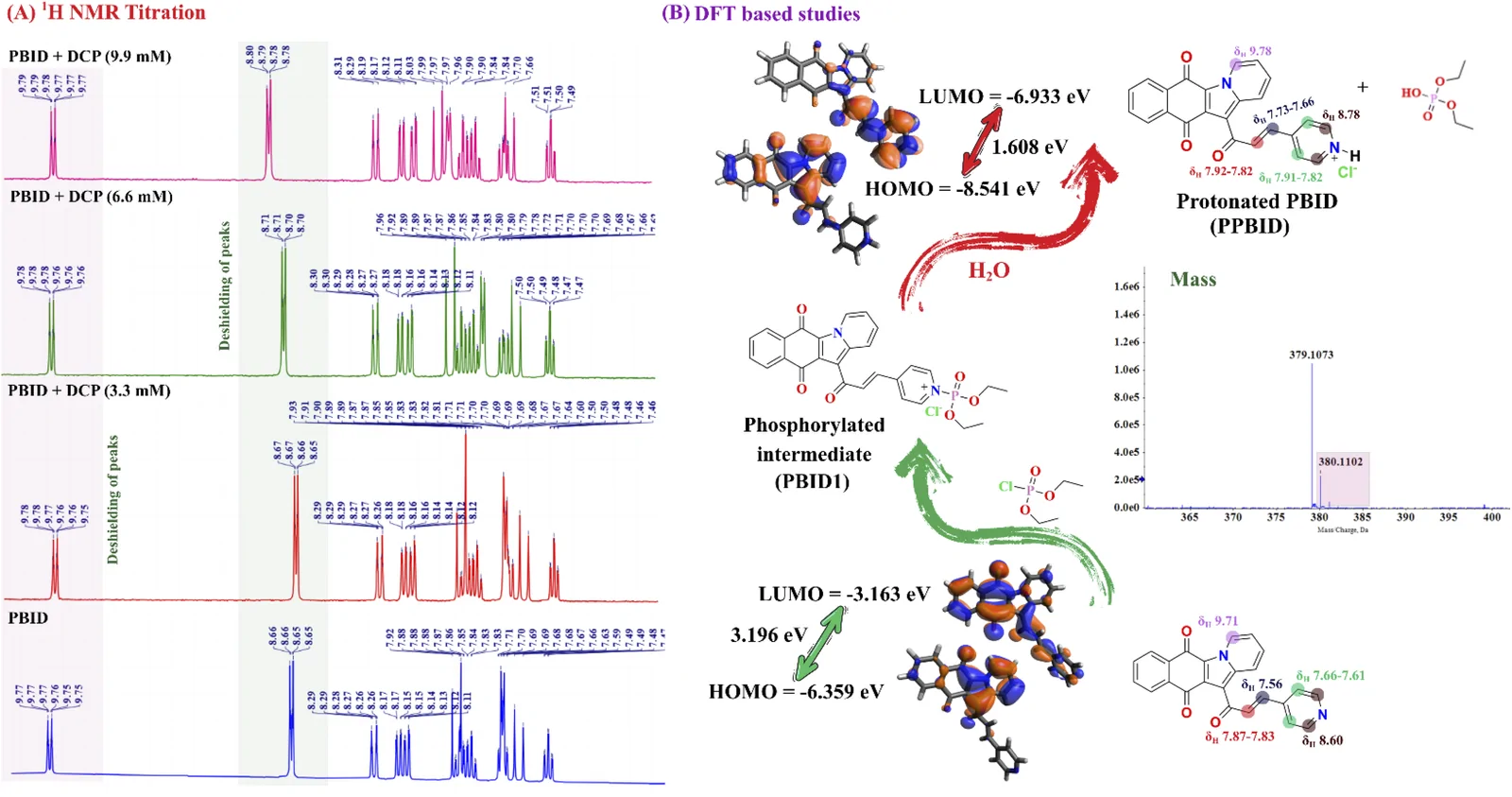
Proposed sensing mechanism validated by (A) ^1^H NMR titration and (B) DFT based calculations.

To investigate the electronic changes associated with the proposed interaction of PBID and DCP, ground state DFT calculations were performed for PBID and its proposed reaction product. The calculated HOMO–LUMO energy gap decreased from 3.196 eV for PBID to 1.608 eV for the proposed product. This reduction was accompanied by substantial stabilisation of both frontier orbitals, particularly the LUMO, whose energy decrease from −3.163 to −6.933 eV. Furthermore, the spatial distributions of the frontier molecular orbitals (Fig. 7B) suggest changes in orbital localisation following the proposed reaction, indicating a modification in the electronic structure of the probe. The HOMO/LUMO plots showed where electron density lies. If the HOMO is on the indolizine–naphthoquinone donor and the LUMO moves onto the acryloyl-pyridine/pyridinium arm after reaction, that confirms charge transfer toward the pyridine and supports the claim that the pyridine nitrogen is the recognition site. Such electronic redistribution may influence the charge-transfer characteristics and photo physical behaviour of the system. However, the reduction in the ground-state HOMO–LUMO gap does not directly explain the experimentally observed fluorescence quenching, since fluorescence emission depends on excited-state properties, radiative and non-radiative relaxation processes. Therefore, the represent DFT results are interpreted as supporting evidence of electronic-structure changes associated with the proposed sensing reaction, rather than definitive proof of the fluorescence-quenching mechanism (Fig. 7). The formation of the protonated complex was further supported by mass spectrometry, where a peak for PBID–DCP complex was observed at *m*/*z* 380.1102 [M + H]^+^, closely matching the calculated value of *m*/*z* 380.1112 [M + H]^+^.

## 4. Real time applications

### 4.1 Solid state fluorescence and latent fingerprinting

Real-time sensing of analytes has emerged as a crucial tool in the field of chemical and environmental monitoring.^33^ For this purpose, the solid-state emission of PBID was first examined under different illumination conditions. The probe displayed vibrant red under day light, and a pitch and bright orange color under short (254 nm) and long UV light (365 nm), respectively (Fig. 8A). These distinct color changes under different illumination conditions indicate the strong solid-state emissive behaviour of PBID and support its potential use in visual sensing applications.

**Fig. 8:**
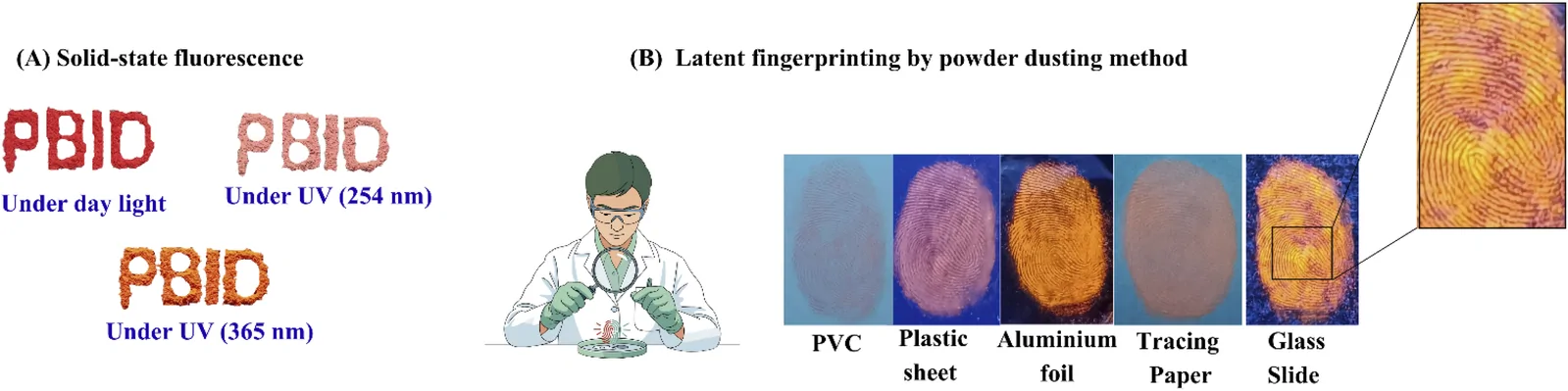
(A) Solid state fluorescence and (B) latent fingerprinting by powder dusting method.

After evaluating the solid-state color variation of PBID, its potential application in latent fingerprint (LFP) visualization was explored.^59^ Fingerprint development was carried out on different substrates, including polyvinyl chloride (PVC), glass slides, aluminium sheets, tracing paper and plastic sheets using the powder dusting method (Fig. 8B). The presence of sweat and sebaceous components in fingerprint residues promoted the adherence of PBID powder to the ridge deposits. Upon uniform dusting of PBID powder over the fingerprint-bearing surfaces, clear and well-defined ridge patterns were observed. The fingerprint images were obtained without any additional post-treatment, thereby simplifying the procedure and reducing the processing time. These fluorescence imaging results demonstrate the versatility of PBID and support its potential applicability in forensic fingerprint visualization.

### 4.2 Solution and solid phase recognition

Real-time sensing of analytes has emerged as a crucial tool in the field of chemical and environmental monitoring.^33^ In the present study, the synthesized probe PBID demonstrated rapid recognition of DCP within 15 s in the solution phase (MeOH). Methanolic solution of PBID exhibited a distinct orange emission under UV light at 365 nm, which changed to lavender upon DCP addition (Fig. 9A). The interaction of other analytes with PBID including TEP, TBP, DMMP, DCMP, DEPA, Na_2_HPO_4_, KH_2_PO_4_, glyphosate, acephate, isoproturon, paraxon, ethion, chlorthioanil, chlorpyrifos and azamethiphos didn't show any significant color change. A schematic representation below illustrates the selective solution phase recognition of DCP by PBID.

**Fig. 9:**
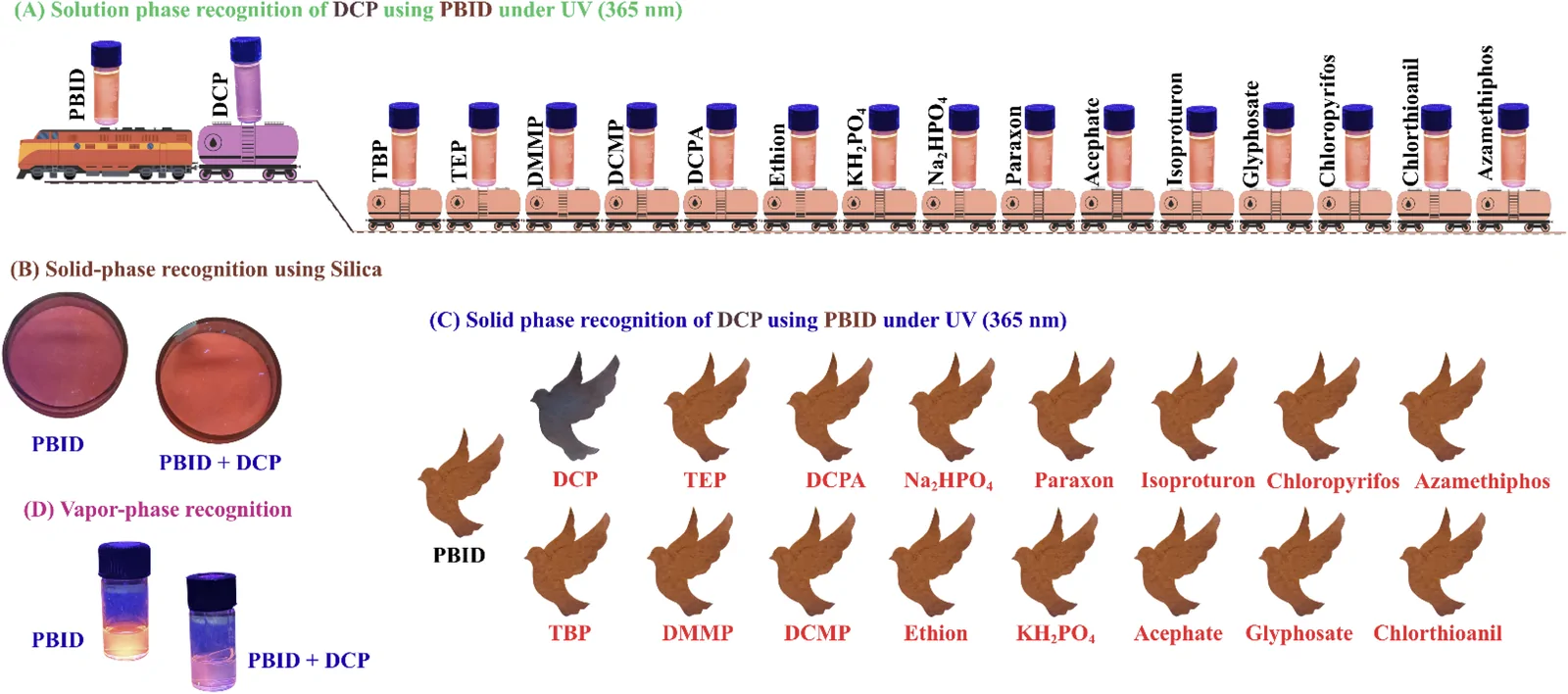
(A) Solution phase; (B) impregnated silica for solid phase recognition; (C) solid phase recognition of DCP under UV light (365 nm) and (D) vapour phase sensing of DCP in methanolic solution of probe.

Following the successful recognition of DCP in the solution phase, the sensing ability of the probe was further evaluated in the solid phase. The solid-phase recognition capability of PBID was also evaluated using silica gel as a support medium and the change was visualized under UV light (365 nm). Upon exposure to DCP, PBID loaded silica showed an intermediate and distinct color change, indicating effective interaction between PBID and DCP in the solid state (Fig. 9B). The rapid fluorescence response on silica gel further extends the practical applicability of PBID for simple, on-site detection of DCP using solid-phase sensing platform.

For evaluating the solid-state responsiveness, a bird-shaped paper test strips impregnated with PBID were fabricated and used for DCP detection. PBID impregnated strips displayed orange emission under UV light at 365 nm, which changed to greyish purple upon exposure to DCP (Fig. 9C). In contrast, no noticeable color change was observed in the presence of other analytes. These observations highlight the excellent selectivity and practical applicability of the developed probe for solid phase detection of DCP under UV light (365 nm).

### 4.3 Vapour phase detection

After successful demonstration of PBID in solid and solution phase DCP recognition, the probe was utilized for vapour-phase detection. This approach is considered more convenient and advantageous over conventional solution-based sensing because of its direct applicability and operational simplicity.^20,45,60^ To evaluate the vapour-phase sensing ability of PBID toward DCP, a sealed glass-vial setup was designed. The outer vial contained a methanolic solution (5 mL) of PBID (10^−3^ M), while a smaller vial containing DCP solution (10^−2^ M) was carefully placed inside the airtight system to allow DCP vapours to diffuse into the PBID solution. Upon exposure to DCP vapours, the emission behavior of PBID changed distinctly from orange to lavender emission under UV light at 365 nm, indicating efficient vapour-phase recognition of DCP (Fig. 9D).

### 4.4 Solid-state reversibility

The ability of PBID to reversibly sense DCP in solution phase has already been established. To evaluate its reversibility in the solid-state, solid-state reversibility experiments were performed. For this purpose, umbrella-shaped paper strips were coated with PBID solution (10^−3^ M) and air-dried under ambient conditions. The prepared test strips exhibited orange emission under UV light (365 nm) and turned to greyish-purple upon DCP (0.01 M) exposure. Interestingly, the original orange emission of PBID revived after treating DCP-exposed strip with triethylamine (TEA) (Fig. 10A). The solid-phase reversibility of PBID further strengthens the 3Rs' principle for the developed methodology.

**Fig. 10:**
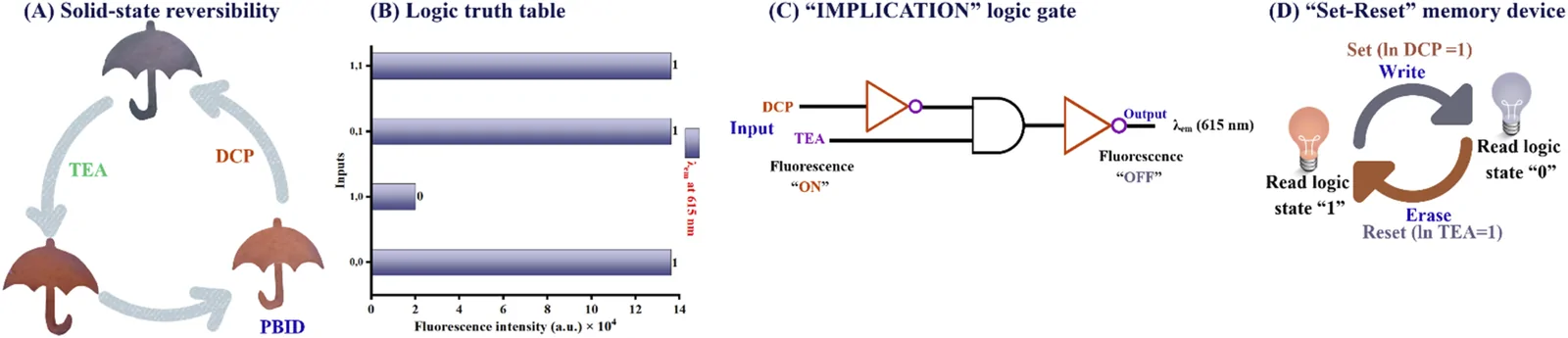
(A) Solid state reversibility; (B) logic truth table; (C) “IMPLICATION” logic gates and (D) molecular devices.

#### 4.4.1 IMPLICATION logic gate and set-reset memory unit

Encouraged by the reversible sensing behavior of PBID toward DCP and TEA, a molecular logic gate was constructed. The optical responses of chemosensors can be effectively interpreted by applying Boolean algebra and molecular logic operations to the observed signals (Fig. 10B). The importance of logic gates in chemical sensing lies in its ability to mimic molecular level information processing through controlled optical responses. A wide range of chemically driven molecular switches can be developed using the distinct color change associated with PBID upon DCP exposure. In the present study, two chemical inputs, DCP (ln 1) and TEA (ln 2) were employed, while the fluorescence intensity at 615 nm was observed as the output signal (Fig. 10C). Here, “1” and “0” represent the “ON” and “OFF” states, respectively. Upon addition of DCP (ln 1 = 1 and ln 2 = 0), the fluorescence quenching was observed resulting in an “OFF” state (output = 0). In contrast, all other input combinations including (1,1), (0,0) and (0,1) resulted into a “ON” fluorescence state (output = 1). A corresponding truth table was constructed using the observed states, resulting in an “IMPLICATION” molecular logic gate operation, which can be represented by a combination of NOT and AND logic functions. Thus, the reversible “ON–OFF–ON” fluorescence modulation demonstrates the potential of PBID as a smart molecular switch for advanced chemical sensing and information processing applications.

Similarly, a molecular memory system based on “Set–Reset” and “Read–Write–Erase” operations was also constructed using the fluorescence state associated with PBID on subsequent addition of DCP and TEA (Fig. 10D). In this system, the binary outputs “1” and “0” assigned as fluorescence “ON” and “OFF” state, respectively. The fluorescence was quenched due to disruption of electronic conjugation pathway, representing the “Write” or “Set” operation and switching the system to the “OFF” state. Interestingly, subsequent addition of TEA restored the original emission intensity by reversing the PBID–DCP interaction, corresponding to “Erase” or “Reset” process. The fluorescence intensity recorded at each stage serve as the “READ” function of the molecular memory device. The reversible “ON–OFF–ON” fluorescence switching demonstrates the potential of PBID as a rewritable molecular switch for chemical information processing and molecular memory applications.

### 4.5 Environmental samples analysis

Following the qualitative examination of probe, quantitative studies were performed to determine the concentration of DCP in environmental samples (water and soil). Diverse water samples including Ganga River water (GW), submersible water (SW), tap water (TW) and drinking water (DW), and soil samples comprising sandy loam (SL), clay loam (CL), black soil (BS) and sandy soil (SS) were analyzed. To evaluate the analytical performance of PBID, known concentration of DCP (20 & 40 µM) and 60 µM was spiked in water samples and soil samples, respectively. Recovery values greater than 90% were observed for all tested samples mentioned in Table S2, supporting the analytical reliability of the developed probe in environmental monitoring (Fig. S4). These outcomes confirm the reliability and practical utility of the developed probe for the quantitative determination of DCP in the environmental samples.

### 4.6 Smartphone based monitoring of DCP concentration and greenness score

The integration of smartphone camera with advanced computing capabilities has expanded the scope of analytical chemistry in real-time monitoring of potential analytes.^61^ To facilitate simple and field deployable monitoring of DCP, a smartphone assisted colorimetric strategy was developed. PBID displayed a pronounced color change from orange to lavender upon addition of DCP under UV light illumination (365 nm). Increasing concentration of DCP (0–100 µM) was added to PBID solution and the color change was analyzed using Color Picker app installed in smartphone (One Plus Nord 2) and the corresponding RGB (Red, Green and Blue) values were extracted from the captured images (Fig. S5). A linear relationship between the R/B *versus* DCP concentration was observed, demonstrating the applicability of smartphone in the quantitative monitoring of DCP. Furthermore, the environmental impact of the developed analytical method was comprehensively evaluated using the AGREE (Analytical GREEnness) metric, which is based on the twelve principle of green chemistry (Fig. S5).^62^ An overall AGREE score of 0.80 highlights the excellent compliance of developed analytical method with green chemistry guidelines.

## 5. Conclusion

In summary, we have successfully developed a naphthoquinone-integrated pyridyl benzopyridoindole dione (PBID) as a selective fluorometric chemosensor for the nerve agent simulant, DCP. The probe operates through a DCP-triggered phosphorylation of the pyridyl nitrogen, followed by hydrolysis to generate a protonated PBID species, which effectively enhanced the intramolecular charge-transfer (ICT) emission. The DFT calculations revealed changes in the frontier molecular orbital energies and their spatial distributions following the proposed interaction of PBID and DCP, indicating substantial modification of the electronic structure of the probe. These findings provide supporting insight into the possible electronic consequences of the sensing reaction. This mechanism endows PBID with an excellent LOD of 0.67 µM in solution and a rapid response time of 15 s. The practical utility of PBID is demonstrated through its versatile applicability across multiple platforms, including low-cost paper strips for vapour-phase detection, visualization of latent fingerprints with high contrast and resolution, and smartphone-assisted RGB analysis for field-based quantitative monitoring. Furthermore, the integration of PBID into “IMPLICATION” molecular logic gate highlights its potential in advanced molecular computing devices. Successful recovery of DCP from spiked environmental matrices (water and soil) validates its real-world deployability, while the AGREE score of 0.80 confirms that the synthetic and analytical protocols align with green chemistry principles. Collectively, this work establishes PBID as a rare example of a single molecular probe that synergistically combines high-sensitivity chemical sensing, forensic visualization, and information processing, offering a promising blueprint for next-generation multi-functional threat surveillance platforms.

## Author contributions

Anju Ranolia: methodology, investigation, formal analysis, data curation, visualization, writing – original draft; Laxmi Narayan – software, visualisation, reviewing and editing; Anil Duhan – formal analysis, reviewing and editing; Sudhir Kumar: reviewing and editing; Parvin Kumar – formal analysis, reviewing and editing; Devender Singh – formal analysis, reviewing and editing; Ahmad Umar: investigation, formal analysis, data curation, visualization, reviewing and editing; S. Baskoutas: investigation, formal analysis, data curation, visualization, reviewing and editing; Jayant Sindhu: supervision, software, data analysis, conceptualization, project administration, reviewing and editing.

## Conflicts of interest

The authors declare that they have no known competing financial interests or personal relationship that could have appeared to influence the work reported in this paper.

## Supplementary Material

RA-OLF-D6RA08860K-s001

## Data Availability

Data will be made available on request. Supplementary information (SI): additional experimental details, characterization data, and supporting results relevant to the synthesis and sensing studies presented in the manuscript. See DOI: https://doi.org/10.1039/d6ra08860k.
