# Supplementary material for: A pyridyl-benzopyridoindole-dione probe for ultrafast fluorometric selective detection of diethyl chlorophosphate (DCP) nerve agent mimic with latent fingerprint and logic gate applications
Source: RSC Adv. 2026 Nov 9. Online ahead of print. doi: 10.1039/d6ra08860k (PMC13747166; doi:10.1039/d6ra08860k)
Supplement: Supplementary file 1 [file RA-OLF-D6RA08860K-s001.pdf]

Supplementary Information (SI) for RSC Advances.
This journal is © The Royal Society of Chemistry

A Pyridyl-Benzopyridoindole-Dione Probe for Ultrafast Fluorometric Selective Detection of diethyl chlorophosphate (DCP) *Nerve Agent Mimic with Latent Fingerprint and Logic Gate Applications*

Anju Ranolia[1], Laxmi Narayan[2], Anil Duhan[1], Sudhir Kumar[2], Parvin Kumar[3,*], Devender Singh[4], Ahmad Umar[5,6,#], Sotirios Baskoutas[7], Jayant Sindhu[1,*]

[1]Department of Chemistry, Chaudhary Charan Singh Haryana Agricultural University, Hisar-125004, Haryana, India

[2]Department of Bioinformatics and Computational Biology, Chaudhary Charan Singh Haryana Agricultural University, Hisar-125004, Haryana, India

[3]Department of Chemistry, Kurukshetra University, Kurukshetra-136119, Haryana, India

[4]Department of Chemistry, Maharshi Dayanand University, Rohtak-124001, Haryana, India

[5]Department of Chemistry, College of Science and Arts, and STEM Pioneers Training Lab, Najran University, Najran-11001, Kingdom of Saudi Arabia

[6]Department of Materials Science and Engineering, The Ohio State University, Columbus 43210, OH, USA

[7]Department of Materials Science, University of Patras, Patras, Greece

#Adjunct Professor in the Department of Materials Science and Engineering, The Ohio State University, Columbus 43210, OH, USA

*Author to whom correspondence should be addressed:

Dr. Jayant Sindhu: jayantchem@gmail.com; **Prof. Parvin Kumar:** parvinjangra@gmail.com

Supporting Info

Table of contents

SI. No.	Description
**Fig. S1.**	Stokes shift plot and Lippert-Mataga plot
1.	Limit of detection (LOD) and Limit of quantification (LOQ)
**Fig. S2.**	Job's plot
**Fig. S3.**	Photo-stability and Thermal-stability
**Fig. S4.**	Smartphone based assessing of DCP concentration and greenness score
**Table S1**	Comparison of stokes shift in different solvents
**Table S2**	Quantitative analysis and recovery studies of DCP in real water and soil samples
**Figure S5-S7**	Characterization spectra of **PBID**

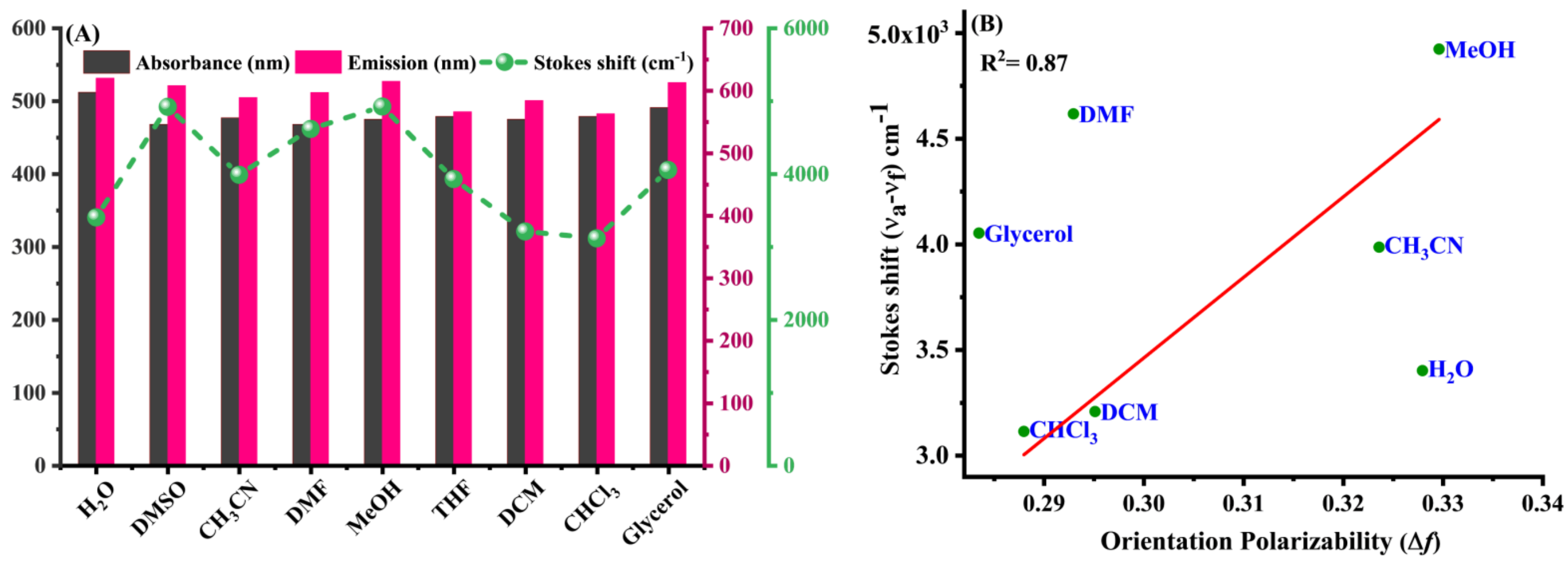

Fig. S1. (A) Stokes shift plot and (B) Lippert-Mataga plot of **PBID**.

1. LOD (Limit of Detection) and LOQ (Limit of Quantification) measurements

The detection and quantification limits were evaluated through fluorescence titration experiments using the equations LOD= 3 σ/k; LOQ= 10 σ/k, where σ denotes the standard deviation of six blank measurements and k corresponds to the slope of the calibration curve.

Linear Equation: $y = -7.03603 \times 10^9 x + 145724$

Here, σ = 1577.868 (The standard deviation of the blank solution; n= 6 measurements); $k = 7.03603 \times 10^9$ (The slope of the concentration titration curve)

LOD = 0.67 μM; LOQ = 2.24 μM.

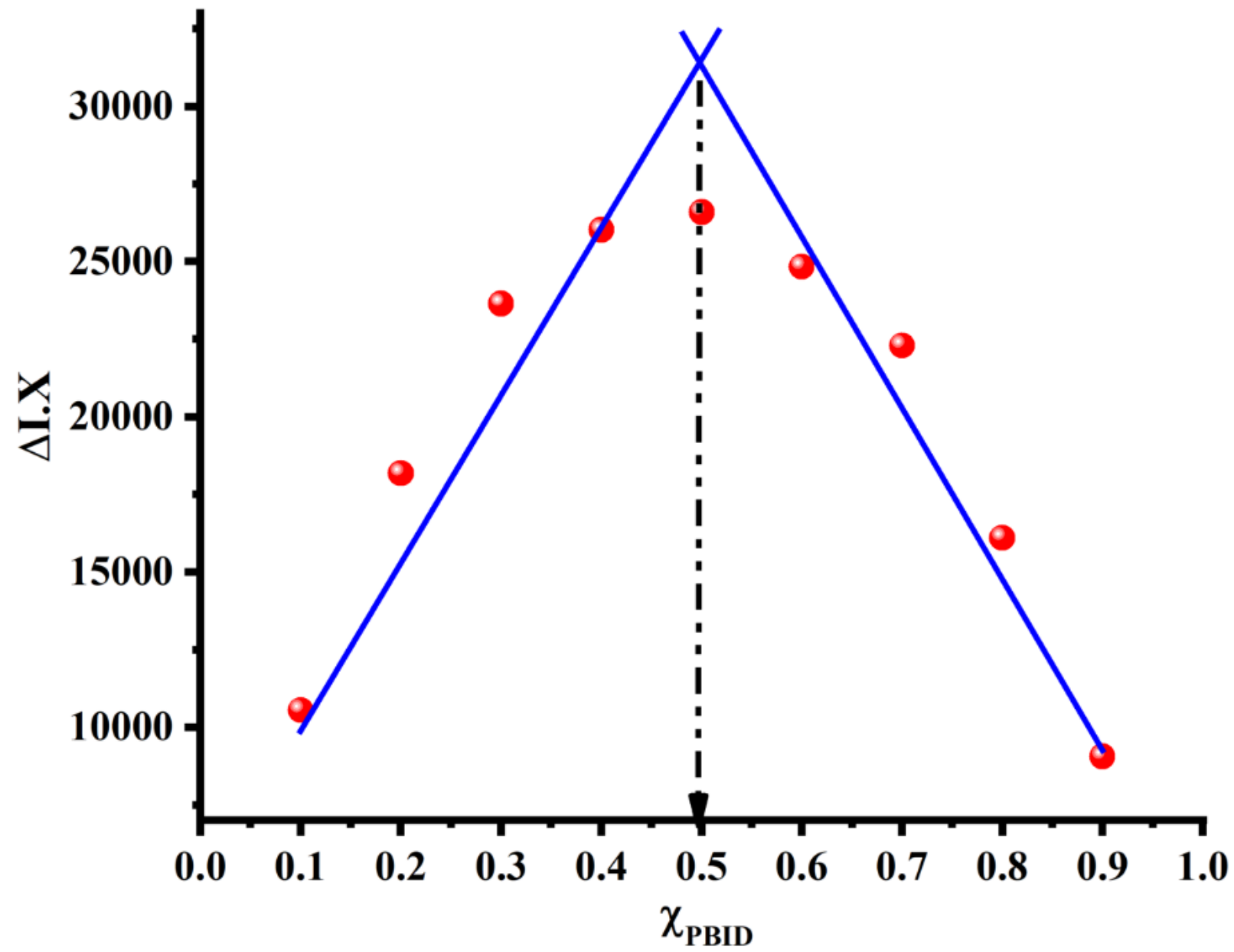

Fig. S2. Job's plot showing 1:1 binding stoichiometry between **PBID** and DCP.

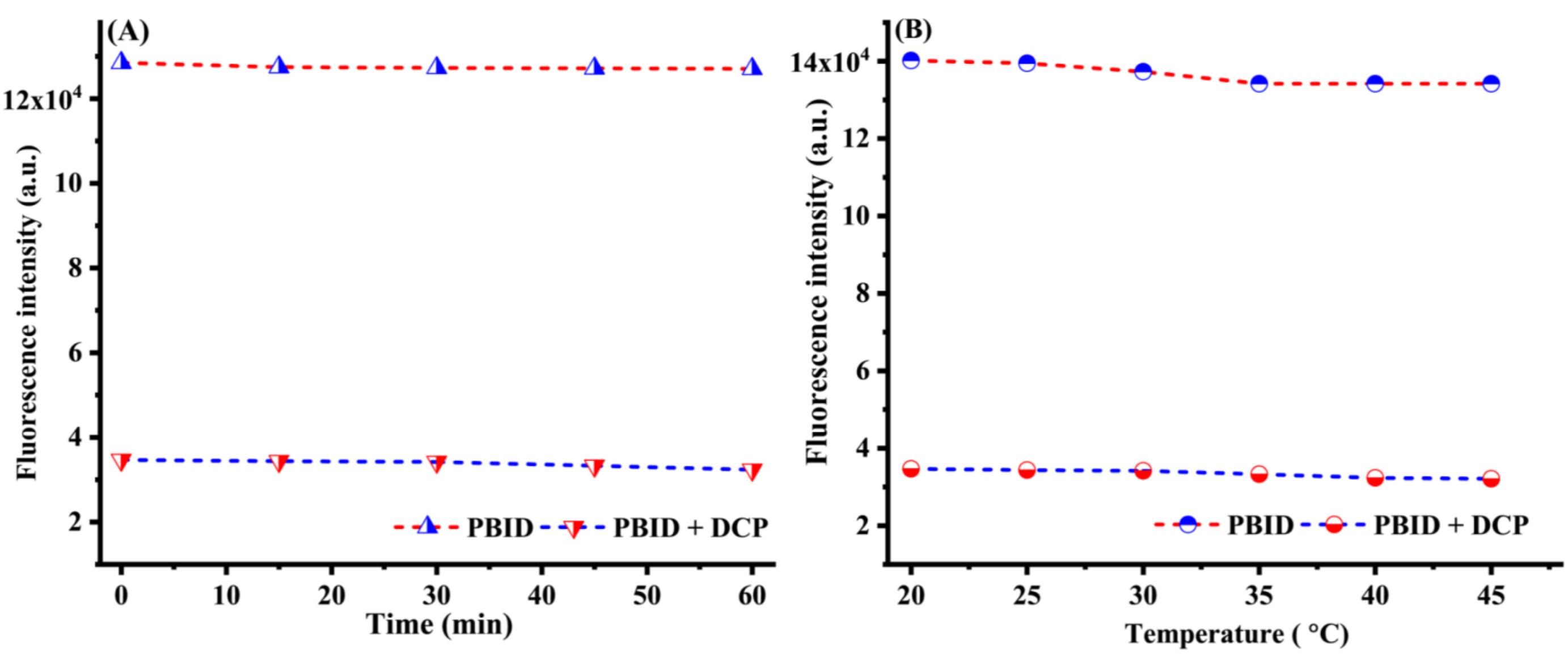

Fig.S3. (A) Photo-stability and (B) Thermal-stability of **PBID** and **PBID**-DCP.

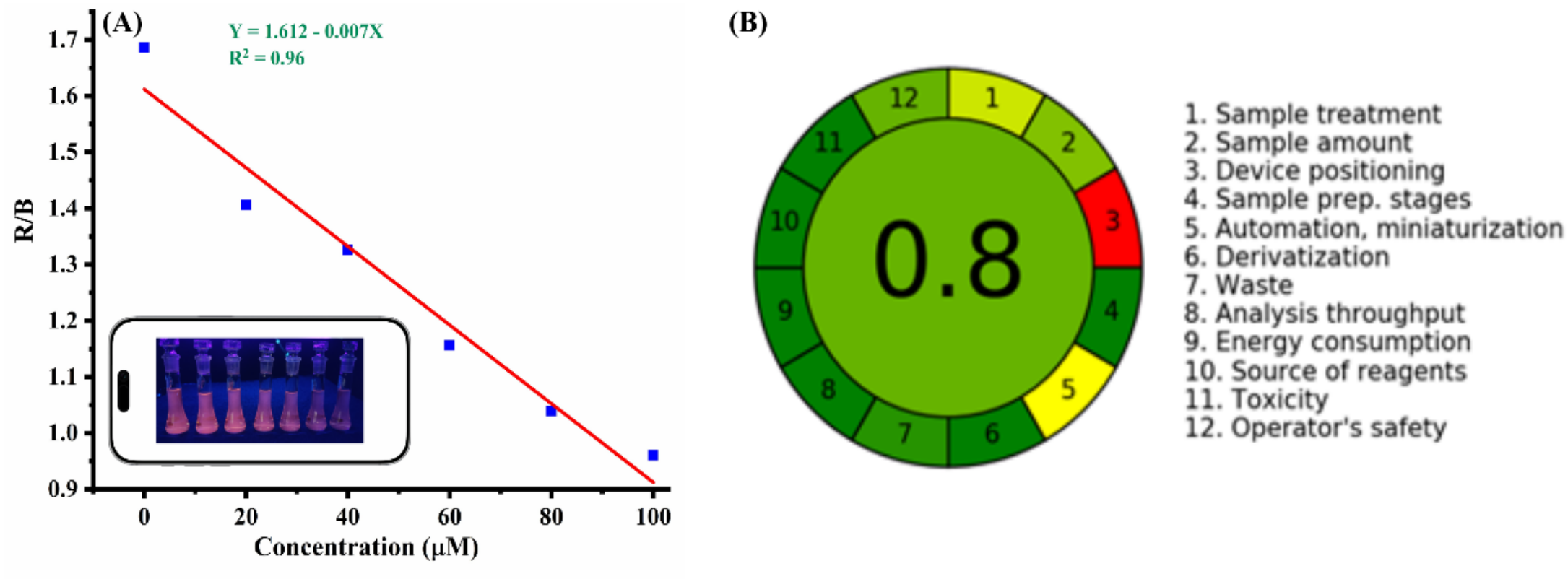

Fig.S4. (A) Smartphone based assessing of DCP concentration (0-100 μM) and (B) greenness score for utilizing probe **PBID**.

Table S1: Stoke shift and Lippert-Mataga plot of **ABID** and **PBID** with varying solvent polarity

Solvents	Compound code	Absorbance (nm)	Absorbance (cm^{-1})	Emission (nm)	Emission (cm^{-1})	Stokes shift (cm^{-1})
H_2O	ABID	506	19762.84	594	16835.01	2927.82
	PBID	512	19531.25	620	16129.03	3402.21
DMSO	ABID	479	20876.82	604	16556.29	4320.53
	PBID	468	21367.52	608	16447.37	4920.15
CH_3CN	ABID	474	21097.04	589	16977.93	4119.11
	PBID	477	20964.36	589	16977.93	3986.43
DMF	ABID	479	21008.40	592	16891.89	4116.51
	PBID	468	21367.52	597	16750.42	4617.10
MeOH	ABID	470	21276.59	613	16313.21	4963.38
	PBID	475	21052.63	615	16260.16	4923.59
DCM	ABID	474	21231.42	565	17699.11	3532.30
	PBID	479	20876.82	566	17667.84	3208.98
THF	ABID	471	21097.04	563	17761.98	3335.05
	PBID	475	21052.63	584	17123.29	3929.34
$CHCl_3$	ABID	473	21141.64	559	17889.08	3252.56
	PBID	479	20876.82	563	17761.99	3114.83
Glycerol	ABID	-	-	-	-	-
	PBID	491	20366.59	613	16313.21	4053.38

Table S 2: Quantitative analysis and recovery studies of DCP in real water and soil samples

Analyte	Water Samples	River water (Ganga)			Submersible water			Tap water			Distilled water		
DCP Concentration	Spiked (μM)	Found (μM) ± SD	Recovery (%)	RSD (%, n=3)	Found (μM) ± SD	Recovery (%)	RSD (%, n=3)	Found (μM) ± SD	Recovery (%)	RSD (%, n=3)	Found (μM) ± SD	Recovery (%)	RSD (%, n=3)
	20	19.49 ± 0.12	97.45	0.62	19.58 ± 0.22	97.90	1.12	19.58 ± 0.15	97.90	0.77	19.71 ± 0.31	98.55	0.57
	40	39.36 ± 0.31	98.40	0.79	39.60 ± 0.31	99.00	0.78	39.60 ± 0.19	99.00	0.48	39.85 ± 0.41	99.63	1.03
	Soil Samples	Sandy loam soil			Black soil			Clay loam soil			Sandy soil		
DCP Concentration	Spiked (μM)	Found (μM) ± SD	Recovery (%)	RSD (%, n=3)	Found (μM) ± SD	Recovery (%)	RSD (%, n=3)	Found (μM) ± SD	Recovery (%)	RSD (%, n=3)	Found (μM) ± SD	Recovery (%)	RSD (%, n=3)
	60	58.55 ± 0.51	97.58	0.87	58.23 ± 0.62	97.05	1.06	59.07 ± 0.55	98.45	0.93	59.56 ± 0.45	99.26	0.76

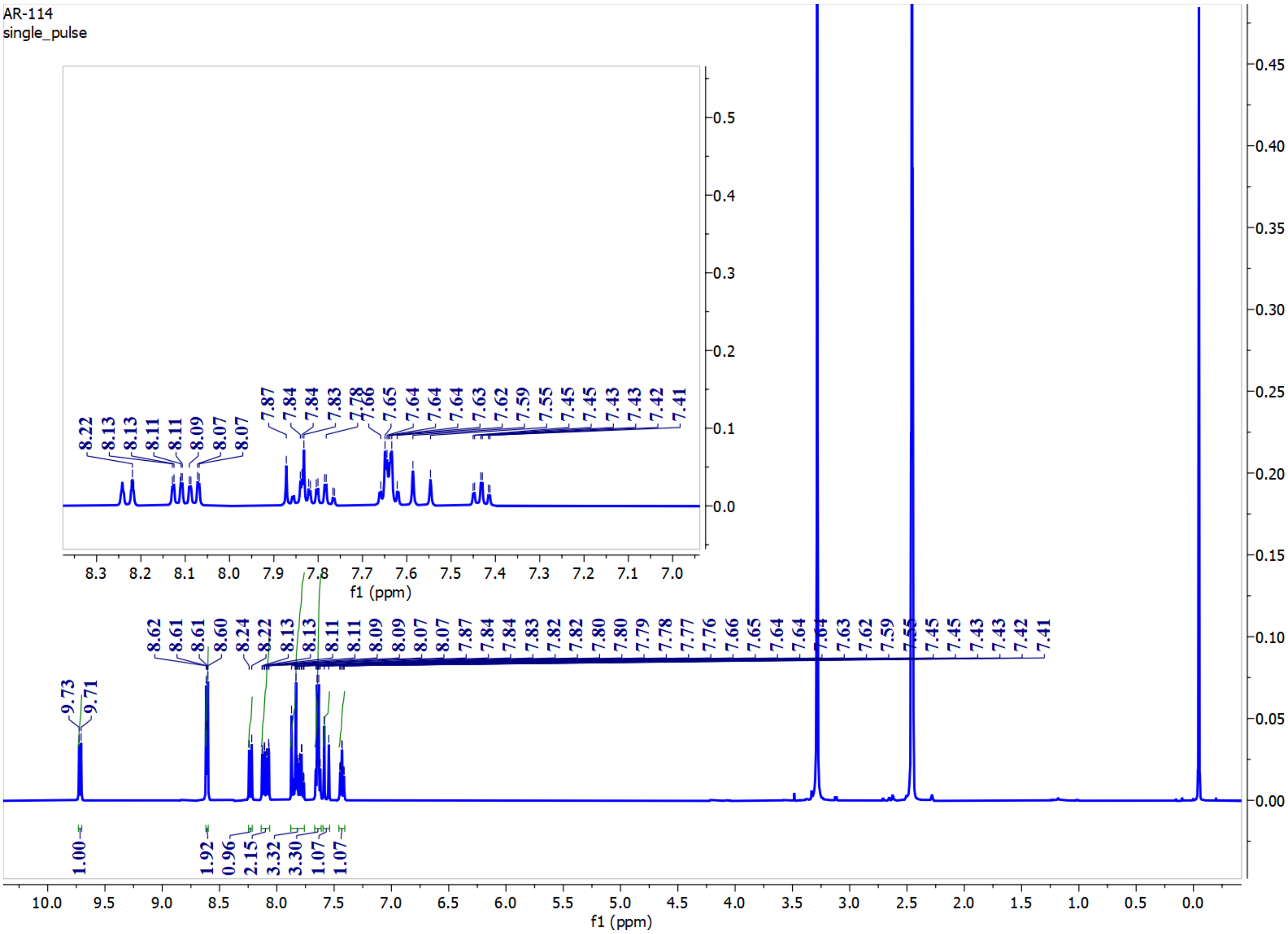

Figure S5: ^{1}H NMR spectra of **PBID**.

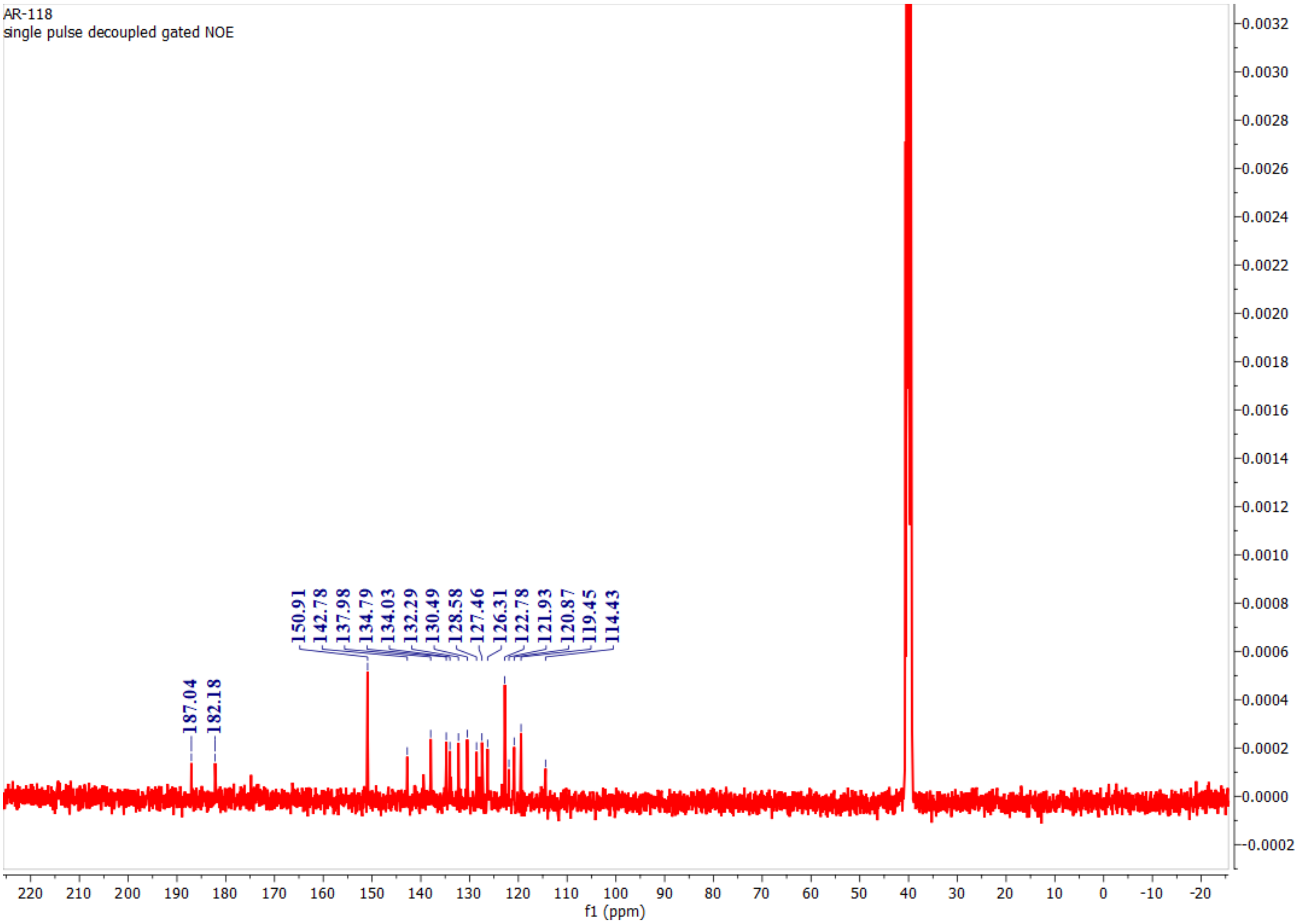

Figure S6: ^{13}C- NMR spectra of **PBID**.

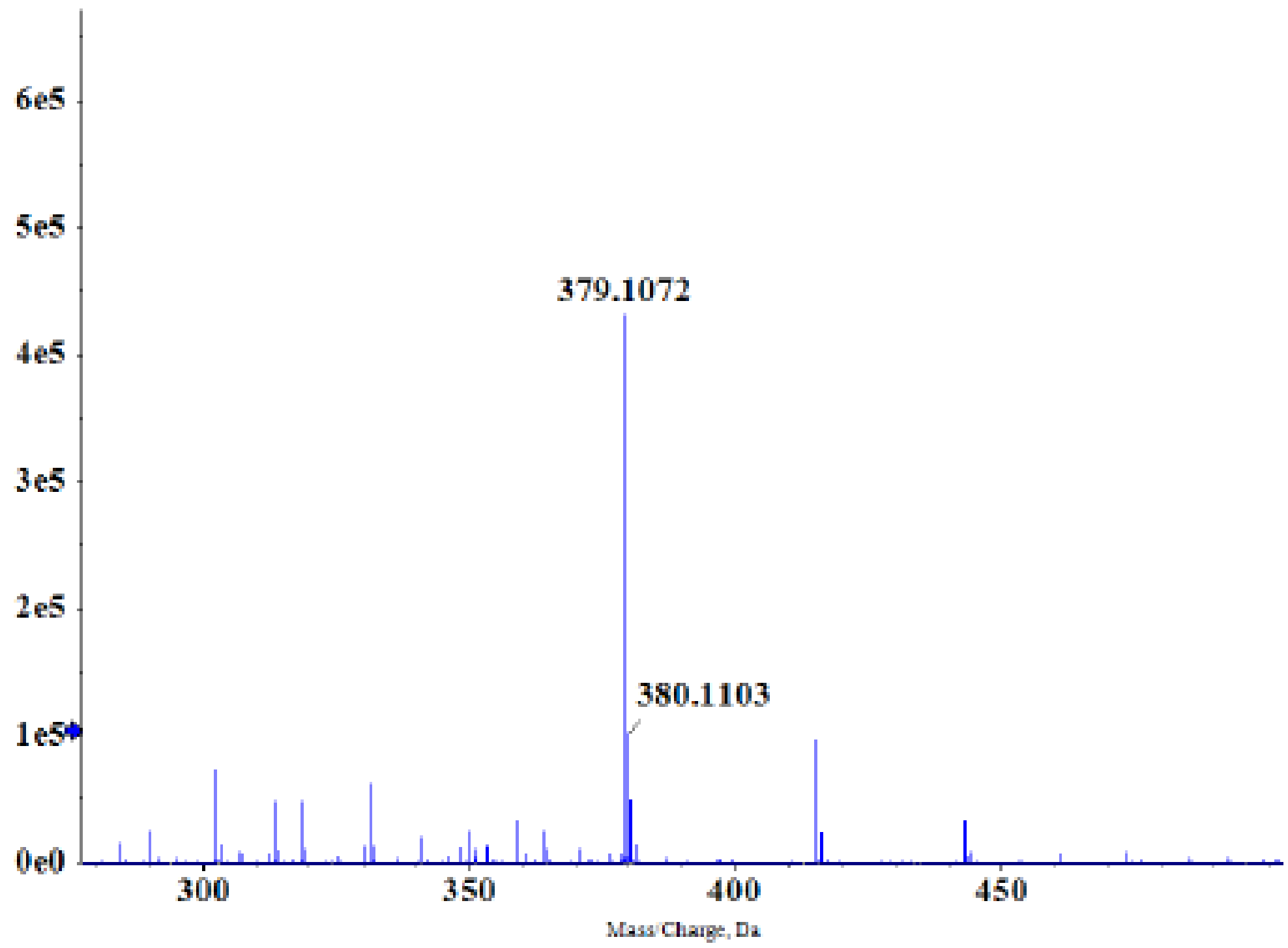

Figure S7: Mass Spectra of **PBID**.

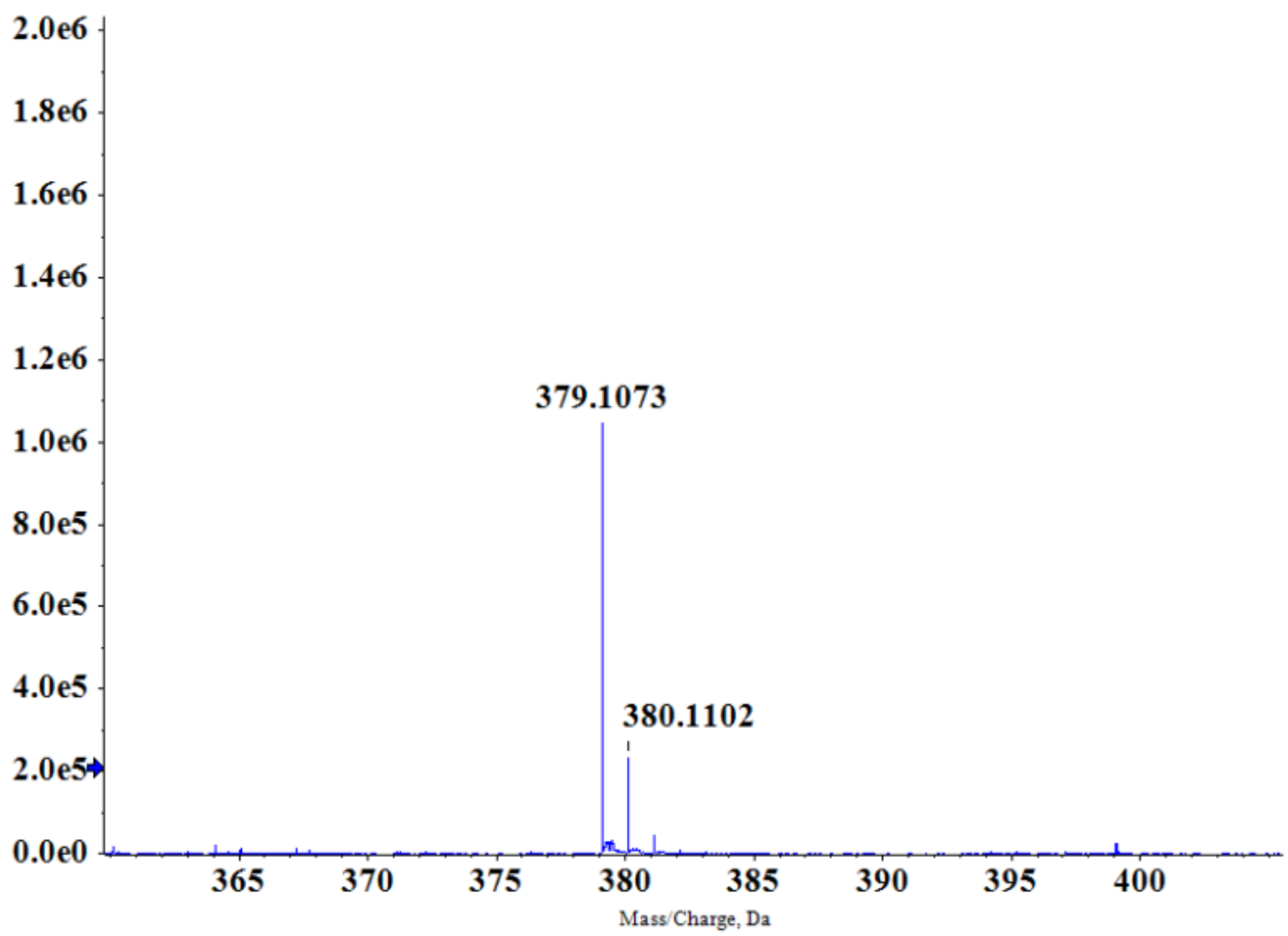

Figure S8: Mass Spectra of **PBID-DCP** complex.
